# Aerobic and sequential anaerobic fermentation to produce xylitol and ethanol using non-detoxified acid pretreated corncob

**DOI:** 10.1186/s13068-014-0166-y

**Published:** 2014-11-23

**Authors:** Ke-Ke Cheng, Jing Wu, Zhang-Nan Lin, Jian-An Zhang

**Affiliations:** Institute of Nuclear and New Energy Technology, Tsinghua University, Beijing, 100084 P.R. China

**Keywords:** Bioconversion, Corncob, Fermentation, Microbial Growth, Inhibitor

## Abstract

**Background:**

For economical bioethanol production from lignocellulosic materials, the major technical challenges to lower the production cost are as follows: (1) The microorganism should use efficiently all glucose and xylose in the lignocellulose hydrolysate. (2) The microorganism should have high tolerance to the inhibitors present in the lignocellulose hydrolysate. The aim of the present work was to combine inhibitor degradation, xylitol fermentation, and ethanol production using a single yeast strain.

**Results:**

A new process of integrated aerobic xylitol production and anaerobic ethanol fermentation using non-detoxified acid pretreated corncob by *Candida tropicalis* W103 was proposed. *C. tropicalis* W103 is able to degrade acetate, furfural, and 5-hydromethylfurfural and metabolite xylose to xylitol under aerobic conditions, and the aerobic fermentation residue was used as the substrate for ethanol production by anaerobic simultaneous saccharification and fermentation. With 20% substrate loading, furfural and 5-hydroxymethylfurfural were degraded totally after 60 h aerobic incubation. A maximal xylitol concentration of 17.1 g l^-1^ was obtained with a yield of 0.32 g g^-1^ xylose. Then under anaerobic conditions with the addition of cellulase, 25.3 g l^-1^ ethanol was produced after 72 h anaerobic fermentation, corresponding to 82% of the theoretical yield.

**Conclusions:**

Xylitol and ethanol were produced in *Candida tropicalis* W103 using dual-phase fermentations, which comprise a changing from aerobic conditions (inhibitor degradation and xylitol production) to anaerobic simultaneous saccharification and ethanol fermentation. This is the first report of integrated xylitol and ethanol production from non-detoxified acid pretreated corncob using a single microorganism.

## Introduction

Fuel ethanol, as a substitute for liquid petroleum fuels, is regarded as one of the important contributors to the reduction of CO_2_. Currently, fuel ethanol has primarily been produced using sugars from crops such as corn, sugarcane, and wheat. However, using crops for fuel ethanol production endangers the food supply, stimulating a demand for advanced technology which can produce ethanol from non-food materials such as waste agricultural and forest residues. Lignocellulosic materials comprise 30 to 45% glucan and 20 to 35% xylan, which, after pretreatment and enzymatic hydrolysis, can be converted to glucose and xylose, respectively. The ethanol production process using lignocellulosic materials will not be economically viable if only the glucose present in the hydrolysate is converted to ethanol [1,2]. However, a traditionally used microorganism for ethanol fermentation, *Saccharomyces cerevisiae*, can only metabolite glucose to ethanol, which makes ethanol production from lignocellulosic materials lacking in competitiveness compared with that from food-based ethanol. Although recent work has succeeded in constructing an efficient xylose metabolic pathway in robust industrial *Saccharomyces cerevisiae* strains, the resulting strains still lacked sufficient inhibitor tolerance for efficient ethanol production using lignocellulosic hydrolysate. Many xylose-fermenting yeasts, such as *Pichia stipitis*, *Candida shehatae*, and *Pachysolen tannophilus*, have been investigated for their use in ethanol production from xylose. However, these microorganisms have much lower ethanol yields and require strict aeration conditions, which limits their use in industrial ethanol production. Some bacteria have the ability to metabolite xylose under anaerobic or aerobic conditions, converting sugars to many products in which ethanol is only a minor product [3-8]. One possibility for addressing this problem is the integrated production of other high-value xylose-based products by means of a biorefinery. The xylan in lignocellulose can be used for high-value-added xylitol production. Further, the glucan residue can be used to produce ethanol. With this method, the total economic viability of the integrated process is better than that with ethanol as the sole product because the price of ethanol is relatively low [1].

Lignocellulosic materials are plentiful and cheap sources of sugars. However, the conversion of cellulose to glucose is not easy, and some form of pretreatment is required. The objective of the pretreatment is to alter the structure of the lignocellulose to increase cellulose biodegradation using cellulase. Dilute acid pretreatment is a favorable route for hemicellulose hydrolysis and also improves the conversion of cellulose to glucose. This method has been successfully applied to different lignocellulosic materials for ethanol production. However, during the acid pretreatment, large amounts of various inhibitors are produced. These inhibitors mainly include furfural, 5-hydroxymethylfurfural (5-HMF), and acetic acid, and the pretreatment conditions (including temperature, time, or acid concentration) will strongly affect the dosages of these compounds and the resulting toxicity. In some previous investigations, the liquid part of the pretreated substrate was separated and then detoxified to decrease the concentration of inhibitor before the subsequent fermentation. However, detoxification usually leads to increased loss of sugar, which will decrease the product yield. Another problem during the detoxification process is the requirement for additional reagents; these reagents cannot be reused, which significantly increases the waste discharge and production cost. Thus, screening for a highly inhibitor-tolerant microorganism, which can use the hydrolysate directly to obtain desirable product, would be very attractive [9-11].

Corncob, which represents 20% of the weight of the harvested corn and has high glucan and xylan contents, is one of the most abundant lignocellulosic wastes in Northeast China. In this study, a new process of integrated aerobic xylitol production and anaerobic ethanol fermentation using non-detoxified acid pretreated corncob by *Candida tropicalis* W103 is proposed. The results obtained may help to find a highly effective way to produce xylitol and ethanol at the same time, which could potentially be applied in lignocellulosic ethanol production.

## Results

### Growth and fermentation profile of *C. tropicalis*

**Figure 1:**
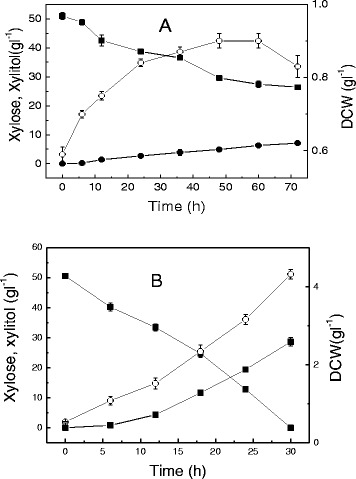
**Time course of xylitol fermentation by** ***Candida tropicalis*** **W103 using xylose. A**: under anaerobic conditions, **B**: under aerobic conditions. Xylose (solid squares), xylitol (solid circles), DCW (open circles). Data presented are average of triplicate experiments; error bars indicate the standard deviations.

*Candida tropicalis* W103 was able to use xylose as the carbon source for cell growth and xylitol production under aerobic or anaerobic conditions (Figure 1). However, *Candida tropicalis* utilized xylose slowly under anaerobic conditions (Figure 1A), and only 48% of the initial xylose was consumed after 72 h of fermentation. The final dry cell weight (DCW) under anaerobic conditions was 0.83 g l^-1^, much lower than the value of 4.32 g l^-1^ under aerobic conditions. The aerobic culture also led to dramatic increases in both xylitol productivity (0.95 g l^-1^ h^-1^) and yield (0.57 g g^-1^ xylose).

**Figure 2:**
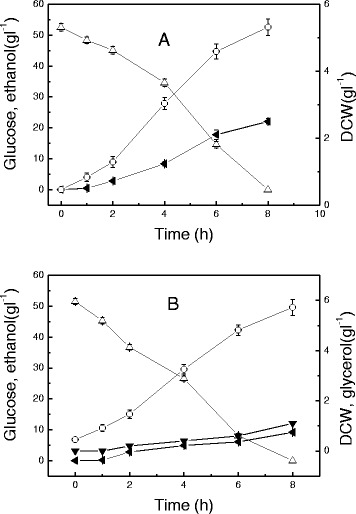
**Time course of ethanol fermentation by** ***Candida tropicalis*** **W103 using glucose. A**: under anaerobic conditions, **B**: under aerobic conditions. Glucose (open triangles), ethanol (solid left-facing triangles), glycerol (solid down-facing triangles), DCW (open circles). Data presented are averages of triplicate experiments; error bars indicate the standard deviations.

When glucose was used as the carbon source, ethanol was produced under aerobic or anaerobic conditions (Figure 2). *C. tropicalis* grew slightly slower under anaerobic conditions than under aerobic conditions. The maximum specific growth rates of *C. tropicalis* in two cases were 0.57 ± 0.04 and 0.53 ± 0.05 h^-1^, respectively. Under aerobic conditions, only 9.2 g l^-1^ ethanol was produced from 51.5 g l^-1^ glucose and 1.1 g l^-1^ glycerol was found in the broth (Figure 2B). In anaerobic conditions, a higher ethanol yield was obtained and the ethanol production from 52.5 g l^-1^ glucose was 22.1 g l^-1^, which corresponded to 82.5% of the theoretical ethanol yield. Thus, *Candida tropicalis* has a different capacity to metabolize glucose and xylose under aerobic or anaerobic conditions.

**Figure 3:**
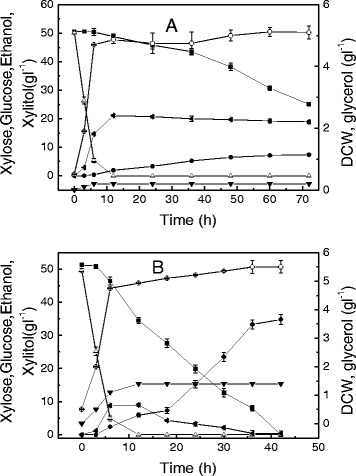
**Sugar fermentation and cell growth of** ***Candida tropicalis*** **W103 using glucose and xylose mixed medium. A**: under anaerobic conditions, **B**: under aerobic conditions. Xylose (solid squares), xylitol (solid circles), glucose (open triangles), ethanol (solid left-facing triangles), glycerol (solid down-facing triangles), DCW (open circles). Data presented are averages of triplicate experiments; error bars indicate the standard deviations.

Considering that glucose and xylose coexist in corncob hydrolysate, *C. tropicalis* W103 using a xylose/glucose mixed medium was investigated under anaerobic and aerobic conditions. Under anaerobic conditions, *C. tropicalis* displayed sequential sugar consumption, first utilizing glucose and then xylose (Figure 3). The maximal growth rate was 0.52 h^-1^ in the mixture medium, similar to that of the glucose-only medium. Only ethanol formation was observed when glucose was used as the substrate. After the glucose was exhausted, about 50% of the initial xylose was consumed, and the xylitol yield was 0.29 g g^-1^ xylose.

Under aerobic conditions, glucose and xylose were consumed at the same time. During the glucose/xylose co-consumption stage, the consumption rate of glucose in the xylose/glucose mixed medium was 7.46 g l^-1^ h^-1^ in 0 to 6 h, much higher than that of xylose (0.81 g l^-1^ h^-1^). A total of 9 g l^-1^ ethanol and 1.4 g l^-1^ glycerol were produced from 49.7 g l^-1^ glucose. However, the ethanol produced in the beginning was volatilized by the end of the fermentation. The xylitol yield under the xylose/glucose mixed medium was higher than that under the xylose medium. This phenomenon can be explained by the idea that the biomass formation in a xylose/glucose mixture medium is mainly attributed to the consumption of glucose and that more xylose will be used for xylitol formation.

From the above study, we found that the xylitol fermentation should be carried out under aerobic conditions and the ethanol yield under aerobic conditions was much lower than that under anaerobic conditions. Thus, a simple aerobic or anaerobic condition is not fit for integrated xylitol and ethanol production using a xylose/glucose mixed medium. To maximize the final product concentration, an aerobic-anaerobic-combined culture was explored further. In this case, xylitol fermentation using a xylose medium (similar to the corncob xylan hydrolysate) was performed under aerobic conditions. After the xylose was consumed, a pulse addition of glucose and a shift to anaerobic culture for glucose fermentation (similar to the corncob glucan hydrolysate) were carried out for ethanol production.

**Figure 4:**
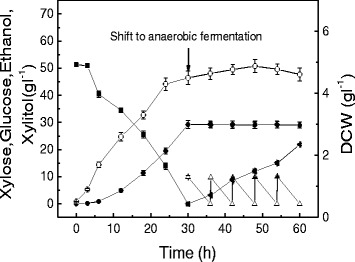
**Anaerobic-aerobic-combined culture using** ***Candida tropicalis*** **W103 for xylitol and ethanol production.** Xylose (solid squares), xylitol (solid circles), glucose (open triangles),ethanol (solid left-facing triangles), DCW (open circles). Data presented are averages of triplicate experiments; error bars indicate the standard deviations.

Similar to a completely aerobic fermentation, the aerobic-anaerobic-combined fermentation from 0 to 30 h had a good xylitol productivity of 0.96 g l^-1^ h^-1^ (Figure 4). After shifting to anaerobic fermentation, glucose was added during 30 to 54 h to simulate glucose release in the enzymatic hydrolysis of glucan. In this way, the ethanol yield could still be maintained at 0.44 g g^-1^ glucose. The final xylitol and ethanol concentrations were 29 and 20.1 g l^-1^, much higher than those under either completely anaerobic or aerobic conditions.

### Inhibitor degradation

**Figure 5:**
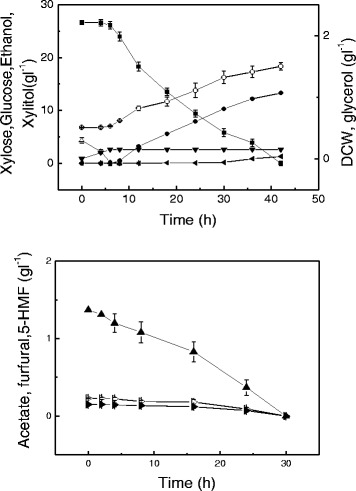
**Inhibitor degradation of** ***C. tropicalis*** **using corncob acid hydrolysate without detoxification.** Xylose (solid squares), xylitol (solid circles), glucose (open triangles), ethanol (solid left-facing triangles), glycerol (solid downward-facing triangles), DCW (open circles), acetate (solid triangles), furfural (open right-facing triangles), 5-HMF (solid right-facing triangles). Data presented are averages of triplicate experiments; error bars indicate the standard deviations.

After acid pretreatment, the corncob hydrolysate is a xylose- and inhibitor-rich medium. The inhibitor degradation of *C. tropicalis* using a hydrolysate without detoxification, which contained 26.64 g l^-1^ xylose, 4.34 g l^-1^ glucose, 0.23 g l^-1^ furfural, 0.15 g l^-1^ 5-HMF, and 1.37 g l^-1^ acetate, was studied under anaerobic and aerobic conditions. Unfortunately, *C. tropicalis* had no ability to degrade the inhibitors under anaerobic conditions and neither ethanol nor xylitol was detected (data not shown). Under aerobic fermentation, low cell growth was observed, and the maximal DCW was only 1.7 g l^-1^ (Figure 5). The maximum specific growth rate of *C. tropicalis* was 0.29 h^-1^, which was 48% lower than that in the medium using pure xylose. Furfural, 5-HMF, and acetate were triggered to degrade after glucose consumption, but prior to xylose. With non-detoxified hydrolysate as the substrate, xylitol formation was slower than that with xylose as the substrate. The final concentration of xylitol was 13.3 g l^-1^ with a yield of 0.5 g g^-1^ xylose and a productivity of 0.32 g l^-1^ h^-1^.

### Combined xylitol and ethanol production

**Figure 6:**
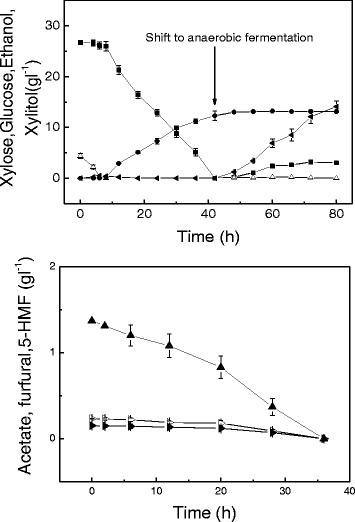
**Xylitol and ethanol combined production using pretreated corncob at a solids loading of 10%.** Xylose (solid squares), xylitol (solid circles), glucose (open triangles), ethanol (solid left-facing triangles), acetate (solid triangles), furfural (open right-facing triangles), 5-HMF (solid right-facing triangles). Data presented are averages of triplicate experiments; error bars indicate the standard deviations.

To examine if the solids (glucan and lignin) containing hydrolysate can be used directly in xylitol and ethanol combined production, aerobic inhibitor degradation and xylitol fermentation were carried out using a pretreated corncob at a solids loading of 10%. After the xylose was consumed totally, a shift to anaerobic simultaneous saccharification and ethanol fermentation was performed with cellulase addition (15 filter paper units (FPU)/g substrate). In this case *C. tropicalis* also can degrade acetate, furfural and 5-HMF, and metabolite xylose to xylitol (Figure 6). However, the rate of inhibitor degradation and xylitol production is decreased, probably due to insufficient mass transfer. Furfural and 5-HMF were degraded totally after 36 h aerobic incubation. A maximal xylitol concentration of 13.1 g l^-1^ was obtained with a yield of 0.49 g g^-1^ xylose. Then, under anaerobic conditions with the addition of cellulase, 14.1 g l^-1^ ethanol was produced after 48 h anaerobic fermentation, corresponding to 79.6% of the theoretical yield.

### Effect of substrate loading

Based on the original composition of 33.2% xylan and 41.4% glucan in corncob, the theoretical yields of xylose and glucose after hydrolysis with 10% substrate loading was 37.5 g and 45.9 g, respectively. However, if a higher substrate loading could be applied, it might be possible to achieve a higher sugar concentration. The initial substrate loading is very critical for xylitol and ethanol combined production from non-detoxified corncob hydrolysate because it is related to both the sugar and inhibitor concentrations.

To investigate the effect of substrate loading on xylitol and ethanol combined production, five sets of batch fermentations were carried out by adjusting strictly the initial water content to progressively increase the substrate loading. With the increase of substrate loading, the xylose and inhibitor concentrations increased. The xylitol and ethanol combined production at different substrate loadings is summarized in Table 1. Decreasing the liquid/solid ratio from 8:1 to 4:1 (based on the initial dry corncob), the xylitol yield was markedly reduced, probably due to insufficient mass transfer and higher inhibitor concentration. In this case, the concentrations of furfural and 5-HMF were 0.71 g l^-1^ and 0.49 g l^-1^, respectively. Only 2.3 g l^-1^ glucose in the medium was consumed, and neither ethanol nor xylitol could be detected. Furfural and 5-HMF were not metabolized by the yeast during the fermentation process. However, the enzymatic hydrolysis seemed to be not affected by the inhibitors. At the end of the fermentation, 60.4 g l^-1^xylose and 61.3 g l^-1^glucose were found in the broth.

**Table 1:** **Xylitol and ethanol combined production by** ***Candida tropicalis*** **W103 using pretreated corncob at different substrate loadings**

**Liquid/solid**	**The composition before fermentation**					**The composition after fermentation**					**Xylitol production**		**Ethanol production**	
	**Glu (g l** ^**-1**^ **)**	**Xyl (g l** ^**-1**^ **)**	**Fur (g l** ^**-1**^ **)**	**5-HMF (g l** ^**-1**^ **)**	**Ace (g l** ^**-1**^ **)**	**Glu (g l** ^**-1**^ **)**	**Xyl (g l** ^**-1**^ **)**	**Fur (g l** ^**-1**^ **)**	**5-HMF (g l** ^**-1**^ **)**	**Ace (g l** ^**-1**^ **)**	**Concentration (g l** ^**-1**^ **)**	**Yield (g g** ^**-1**^ **xylose)**	**Concentration (g l** ^**-1**^ **)**	**Yield (g g** ^**-1**^ **glucose)**
8	5.43	33.31	0.18	0.29	1.71	0	3.4	0	0	0	14.7	0.44	19.7	0.42
7	6.20	38.06	0.21	0.34	1.95	0	3.1	0	0	0	16.4	0.43	20.7	0.42
6	7.24	44.41	0.24	0.39	2.28	0	2.8	0	0	0.12	16.0	0.36	23.2	0.43
5	8.69	53.29	0.29	0.47	2.74	0	3	0	0	0.29	17.1	0.32	25.3	0.42
4	10.86	66.61	0.36	0.59	3.42	61.2	66.6	0.59	0.41	2.70	-	-	-	-

The values are the means of three independent samples.

Glu: glucose, Xyl: xylose, Fur: furfural, Ace: acetate.

**Figure 7:**
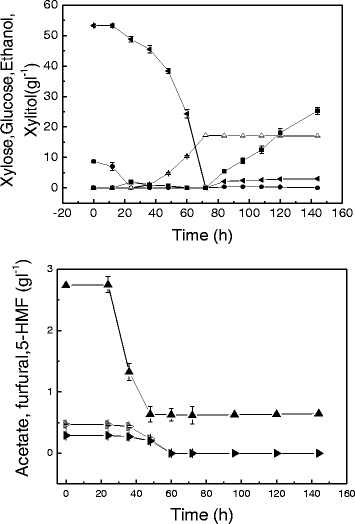
**Xylitol and ethanol combined production using pretreated corncob at a initial liquid/solid 5:1 medium.** Xylose (solid squares), xylitol (solid circles), glucose (open triangles), ethanol (solid left-facing triangles), acetate (solid triangles), furfural (open right-facing triangles), 5-HMF (solid right-facing triangles). Data presented are averages of triplicate experiments; error bars indicate the standard deviations.

Figure 7 displays a typical fermentation profile of *C. tropicalis* fermenting the pretreated corncob at liquid/solid ratio of 5:1, which caused a 12-h lag phase in aerobic xylitol production. Some inhibitors, such as furfural and 5-HMF, were metabolized totally in 60 h. However, acetate could not be metabolized totally and was kept at a low level of 0.29 g l^-1^ after both furfural and 5-HMF were completely degraded at 48 h of fermentation. The final concentration of xylitol was 17.1 g l^-1^ with a yield of 0.32 g g^-1^ xylose. After 72 h, the xylose in the broth was consumed, and the fermentation was then shifted to anaerobic culture with cellulase addition. Another 72 h of anaerobic simultaneous saccharification and fermentation (SSF) was carried out, resulting in the highest ethanol concentration of 25.3 g l^-1^, corresponding to 82% of the theoretical ethanol yield.

The substrate loading of liquid/solid 5:1 resulted in the highest xylitol concentration of 17.1 g l^-1^. However, a lower substrate concentration (liquid/solid 7:1) also gave a favorable result of 16.4 g l^-1^ for the final xylitol concentration. The probable reasons for this were better mass transfer and lower inhibitor concentration. The highest ethanol concentration of 25.3 g l^-1^ was obtained with a substrate loading of liquid/solid 5:1. The maximum ethanol yield from corncob was 0.155 g g^-1^, which was achieved at liquid/solid 8:1. In this process 81.5% of the glucan was hydrolyzed to monomeric glucose and then to ethanol with a yield of 0.42 g g^-1^ glucose.

## Discussion

Many microorganisms are severely inhibited by the toxic compounds generated in the pretreatment process of lignocellulose. Thus, an efficient removal of the inhibitors is a necessary step for the bioconversion process using lignocellulosic material as the feedstock. In the previous studies on dilute acid pretreatment, efforts have been devoted to the optimization of operation parameters such as treatment temperature, time, and acid concentration to improve the yield of sugar and reduce the concentration of toxins by different kinds of detoxification. Many detoxification methods have been applied to alleviate the inhibition of hydrolysate for improving microbial growth. For the xylan hydrolysate part, methods such as overliming, the use of ion exchange resins, or activated charcoal adsorption can remove most of the toxins. For the solid part (mainly glucan and lignin), water washing has been used regularly in the literature. However, these methods either result in considerable loss of sugar or generate massive amounts of wastewater. Another problem during the detoxification process is the requirement for additional operation units, which significantly increases the production cost [12-15].

To avoid the drawbacks in these routine detoxification methods, the development of an inhibitor-tolerant microorganism is considered as a promising method because it has many advantages such as mild conditions, low energy input, and no wastewater release. Currently, many bacteria and fungi have been isolated and identified to have a toxin degradation capacity, but combining the inhibitor degradation with the product fermentation practice using non-detoxified lignocellulosic feedstock has not been demonstrated.

Several strains have been used to ferment xylan acid hydrolysate from various raw materials for xylitol production. Only a few studies have focused on the efficient use of all glucose and xylose in the lignocellulose hydrolysate [16-19]. In a co-culture of *S. cerevisiae* and *Spathaspora arborariae* under oxygen limitation, glucose and xylose in rice hull hydrolysate were converted to ethanol and xylitol, with concentrations of 11 g l^-1^ and 3 g l^-1^, respectively [20]. *C. tropicalis* W103 used in the present study showed good potential for xylitol and ethanol combined production because higher product concentrations and productivity were obtained. Furthermore, compared with the inhibitor concentration in the fermentation medium, *C. tropicalis* W103 was more tolerant to the inhibitors present in the hydrolysates.

In this study, *C. tropicalis* W103 is able to degrade acetate, furfural and 5-hydromethylfurfural, and metabolite xylose to xylitol under aerobic conditions and produce ethanol by SSF under anaerobic conditions. The developed yeast showed many advantages. Xylitol and ethanol combined production using *C. tropicalis* W103 is carried out by changing from aerobic conditions to anaerobic SSF. This process lowered the possibility of contamination by other microbial organisms because only a single inoculation was needed.

The high solids culture model was applied for inhibitor degradation by *C. tropicalis* W103 on the pretreated corncob directly instead of performing inhibitor degradation in the liquid hydrolysate. No cellulose degradation was observed during the *C. tropicalis* W103 culture on hydrolysates containing highly cellulose-rich solids, and thus the cellulose loss could be avoided in the aerobic stage. The process was also preferable because there were no additional operation units, no sugar loss, and no wastewater generation. This is particularly important because the process can combine inhibitor degradation, xylitol fermentation, and ethanol production in the same bioreactor. Finally, the high solids culture model of inhibitor degradation proved to be a fast process: most toxins were degraded within 24 to 48 h, and the consequent xylitol and ethanol production was performed in 48 to 72 h. Because ethanol and xylitol have different boiling points, a simple approach to remove ethanol from the fermentation broth can be carried out by evaporation, and further purification of concentrated xylitol can be done by crystallization.

The disadvantage of the current xylitol and ethanol combined production by *C. tropicalis* is its relative low product concentration compared with solo product production due to limited substrate loading. In Zhang’s report, a bioreactor with a novel helical impeller was designed and applied to the high substrate loading. This stirring system had better performances in terms of saccharification yield, product concentration, and energy cost [21]. This new bioreactor has the potential to be applied in xylitol and ethanol combined production to obtain higher product yield and concentration.

## Conclusions

In this study, a new process of integrated aerobic xylitol production and anaerobic ethanol fermentation using non-detoxified acid pretreated corncob by *Candida tropicalis* W103 was proposed. Its advantages include no additional operation unit, zero wastewater generation, processing on solid pretreated material, and no need for sterilization, all of which can help make the lignocellulosic ethanol industry more cost-effective and environmentally friendly, providing more potential for industrial application.

## Materials and methods

### Raw material

The corncobs were grown in Heilongjiang Province in Northeast China and harvested in 2012. The air-dried material was ground to 2- to 4-mm (5 to 10 mesh) particle size. The corncob particles were dried at 105°C for 4 h and then stored in sealed plastic bags at room temperature for use. The initial composition of the biomass was 2.88 ± 0.04% benzene-ethanol extractives, 41.4 ± 0.31% glucan, 33.2 ± 0.41% xylan, and 18.9 ± 0.33% lignin.

The cellulase used in the enzymatic hydrolysis was Cellulase 0816, which was purchased from Habio Biotech Co. Ltd. in China. The cellulase activity was determined by the method proposed by Ghose, and expressed in filter paper units (FPU) [22].

### Pretreatment

The pretreatment was carried out in 500-ml glass flasks. 20 g of corncobs at a solid-liquid ratio of 1:3 was mixed with dilute acid (0.5% (w/w) H_2_SO_4_ + 1.5% (w/w) H_3_PO_4_) and pretreated in an autoclave at 130°C with a residence time of 60 min.

### Microorganism and fermentation experiments

*Candida tropicalis* W103 was grown on the preculture medium containing 10 g l^-1^ yeast extract, 20 g l^-1^ peptone, and 20 g l^-1^ glucose. The seed cells were prepared in 500-ml flasks containing 100 ml preculture medium. The flasks were incubated at 35°C and 180 rpm for 14 h and subsequently inoculated into the fermentation medium at 10% (v/v). The biomass concentration at the beginning of the fermentation ranged from 0.5 g l^-1^ to 0.53 g l^-1^.

In the growth and fermentation tests of *C. tropicalis*, the prepared xylose or glucose, supplemented with 2 g l^-1^ KH_2_PO_4_, 5 g l^-1^ (NH_4_)_2_SO_4_, 0.5 g l^-1^ MgSO_4_ · 7H_2_O, 1 g l^-1^ peptone, and 5 g l^-1^ yeast extract, was used as the fermentation medium. All anaerobic cultures were performed as batch fermentations with 200-ml working volumes in 500-ml pin plug flasks. The residual oxygen in the medium and head space of the flasks was not flushed out in order to support initial cell growth. The flasks were incubated at 35°C in an orbital shaker with a rotation speed of 100 rpm. Aerobic fermentation experiments were performed with a 100-ml working volume in 500-ml gauze-plugged flasks. The flasks were incubated at 35°C in an orbital shaker with a rotation speed of 160 rpm.

For testing the yeast’s ability to degrade the inhibitors, 140 ml of water was added to the flasks with 20 g pretreated corncob, and the flasks were shaken in a shaker at 30°C and 160 rpm for 15 min. The liquid fraction was recovered by filtration, and the pH was adjusted to 5 using Ca(OH)_2_. The obtained liquid hydrolysate, supplemented with 2 g l^-1^ peptone and 5 g l^-1^ yeast extract, was used as the fermentation medium.

For the xylitol and ethanol combined production, the acid-hydrolyzed corncob containing the remaining solid fraction that did not solubilize during the acid hydrolysis was used directly without sterilization. Water was added strictly to adjust the initial solid-liquid ratio of 1:4 to 1:8. The pH was adjusted to 5 using solid Ca(OH)_2_. The high solid medium, supplemented with 2 g l^-1^ peptone and 5 g l^-1^ yeast extract, was used as the fermentation medium. In the stage involving aerobic cultures for inhibitor degradation and xylitol production, these gauze-plugged flasks were incubated at 35°C and 160 rpm. After shifting to anaerobic simultaneous saccharification and ethanol fermentation, these flasks were sealed with pin plugs and incubated at 35°C and 100 rpm.

#### Analytical methods

The liquid samples were analyzed by HPLC (Shimadzu LC 20A, Japan), equipped with UV and refractive index (RI) detectors. The concentrations of xylose, glucose, acetic acid, glycerol, ethanol, and xylitol were determined using an Aminex HPX-87H column and an RI detector at 65°C with 5 mM H_2_SO_4_ as the mobile phase at 0.8 ml min^-1^. Furfural and 5-HMF were detected using a UV detector at 280 nm. Cell growth was monitored with optical density at 650 nm and converted to dry cell weight (DCW) by a calibration curve.

## Abbreviations

5-HMF: 5-hydroxymethylfurfural
DCW: dry cell weight
FPU: filter paper units
SSF: simultaneous saccharification and fermentation
